# Beyond defense: regulation of neuronal morphogenesis and brain functions via Toll-like receptors

**DOI:** 10.1186/s12929-019-0584-z

**Published:** 2019-11-04

**Authors:** Chiung-Ya Chen, Yi-Chun Shih, Yun-Fen Hung, Yi-Ping Hsueh

**Affiliations:** Institute of Molecular Biology, Academia Sinica, 128, Academia Rd., Sec. 2, Taipei, 11529 Taiwan Republic of China

**Keywords:** Innate immunity, TLR, Neuronal development

## Abstract

Toll-like receptors (TLRs) are well known as critical pattern recognition receptors that trigger innate immune responses. In addition, TLRs are expressed in neurons and may act as the gears in the neuronal detection/alarm system for making good connections. As neuronal differentiation and circuit formation take place along with programmed cell death, neurons face the challenge of connecting with appropriate targets while avoiding dying or dead neurons. Activation of neuronal TLR3, TLR7 and TLR8 with nucleic acids negatively modulates neurite outgrowth and alters synapse formation in a cell-autonomous manner. It consequently influences neural connectivity and brain function and leads to deficits related to neuropsychiatric disorders. Importantly, neuronal TLR activation does not simply duplicate the downstream signal pathways and effectors of classical innate immune responses. The differences in spatial and temporal expression of TLRs and their ligands likely account for the diverse signaling pathways of neuronal TLRs. In conclusion, the accumulated evidence strengthens the idea that the innate immune system of neurons serves as an alarm system that responds to exogenous pathogens as well as intrinsic danger signals and fine-tune developmental processes of neurons.

## Introduction

Organisms have host defense systems, namely adaptive and innate immunity, to tackle pathogen invasion [1]. The adaptive immune system evolved in vertebrates. It is highly specific and can establish long-term immune memory for antigens in specific immune cells, such as B and T lymphocytes [1, 2]. The innate immune system is the first line of defense, and some forms of it exist in most cell types of all species. Phagocytosis and cytokine/chemokine production are two critical elements of innate immunity. Initial phagocytosis by macrophages or other phagocytes triggers production and release of cytokines and/or chemokines. Pathogen-infected cells can also release cytokines/chemokines. Those cytokines and/or chemokines further recruit more immune cells to effectively eliminate foreign pathogens and infected cells [3]. Therefore, unlike the adaptive immune system, the innate immune system responds quickly to danger signals and lacks antigen specificity.

In the innate immune system, cells use a variety of pattern recognition receptors (PRRs) to detect molecules derived from bacteria, viruses, and parasites. Such PRRs include the toll-like receptors (TLRs), C-type lectin receptors (CLRS), NOD-likes receptors (NLRs), RIG-like receptors (RLRs), and AIM2-like receptors (ALRs) [3–5]. The different PRRs recognize divergent pattern molecules and highly diverse PRRs equip the host with the ability to detect various pathogens.

Toll/TLR superfamily is the first to be identified and the best characterized protein family of PRRs. Thus far, the Toll/TLR superfamily has been found in all animals except Phylum Porifera (sponges). In addition to detecting the pathogen-associated molecular patterns (PAMPs) of microorganisms, TLRs also recognize endogenous damage or danger signals (damage-associated molecular patterns, DAMPs), such as self mRNA and DNA derived from dead cells (caused by either apoptosis or other stress) or autophagosomes, miRNA released through the exosomal pathway, and other macromolecules derived from injured tissues [6–15].

Toll, the first reported member of the Toll/TLR superfamily, was discovered in *Drosophila* and was originally identified as a membrane protein determining dorsoventral polarity [16, 17]. Functional studies suggest that most Toll family proteins in the fruit fly are important during embryonic development and that some of them also mediate innate immune responses [18–20]. Interestingly, Tolls recognize neurotrophins to control neuronal survival and death [21, 22]. Toll-6 and Toll-7 also act as adhesion molecules to mediate synaptic partner matching in the *Drosophila* olfactory circuit [23]. The signaling pathways and functions of Tolls in fly development have been revealed and previously reviewed by others [21, 22, 24–27].

There is no evidence to support interaction of TLRs with neurotrophic factor(s) in mammalian brains. Instead, activation of TLRs by PAMPs or DAMPs influences neurogenesis, neuronal differentiation and maturation [5, 28]. TLR deficiency results in abnormal mouse behaviors, such as learning and memory defects and the features of neurodevelopmental disorders. Moreover, immune activation of TLRs at early developmental stages impairs neural development and increases the risk of developing neuropsychiatric disorders, including schizophrenia and autism spectrum disorders [29, 30]. Although peripheral cytokines (e.g. IL-6 and IL-17) were thought to be critical for immune activation-induced abnormalities in brain development and neuropsychiatric disorders [31, 32], evidence (detailed below) suggests that neuronal TLR activation can also influence neuronal morphology and alter brain function. Thus, both DAMPs and PAMPs likely control neuronal morphology via TLR activation. In this article, we focus on the effects and mechanisms of TLRs in neuronal morphogenesis to highlight the non-defense function of the innate immune machinery in neurons.

### Mammalian TLRs and their domain structures

**Fig. 1:**
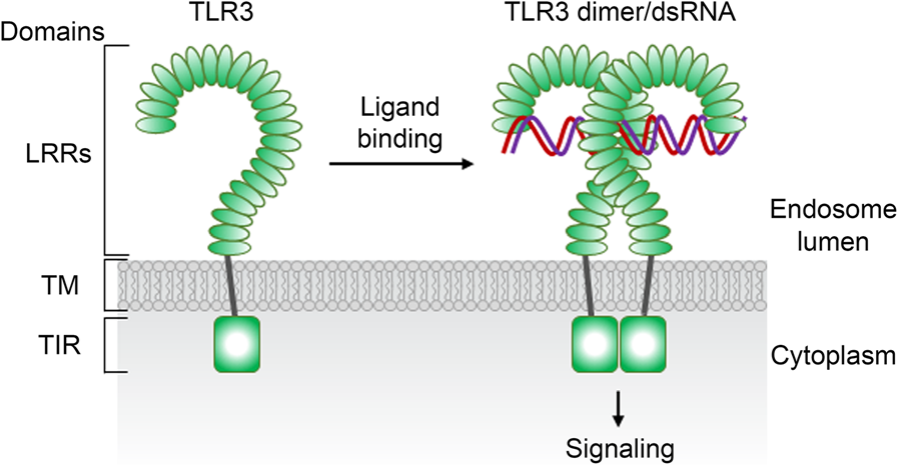
Schematic of the protein domain structure of TLRs. TLR3 is used as an example here. Binding of double-stranded RNA (dsRNA) induces TLR3 dimerization, leading to activation of downstream signaling. LRRs, leucine-rich repeats; TIR, Toll/interleukin-1 receptor; TM, transmembrane domain. Proteolysis to cleave the ectodomain is also involved in TLR3 activation, but it is not indicated here

TLRs contain multiple leucine-rich repeats (LRRs) at the N-terminus, a single transmembrane domain, and a C-terminal Toll/interleukin-1 receptor (TIR) domain. The N-terminal LRRs form a horseshoe-shaped structure that mediates recognition of exogenous and endogenous pattern molecules. The TIR domain binds adaptor molecules and initiates signaling transduction [33, 34]. Though the numbers of LRR vary, the basic structures of different TLRs are similar. Here, we employ TLR3 as an example to show the basic domain organization of TLRs (Fig. 1, Table 1).

**Table 1:**
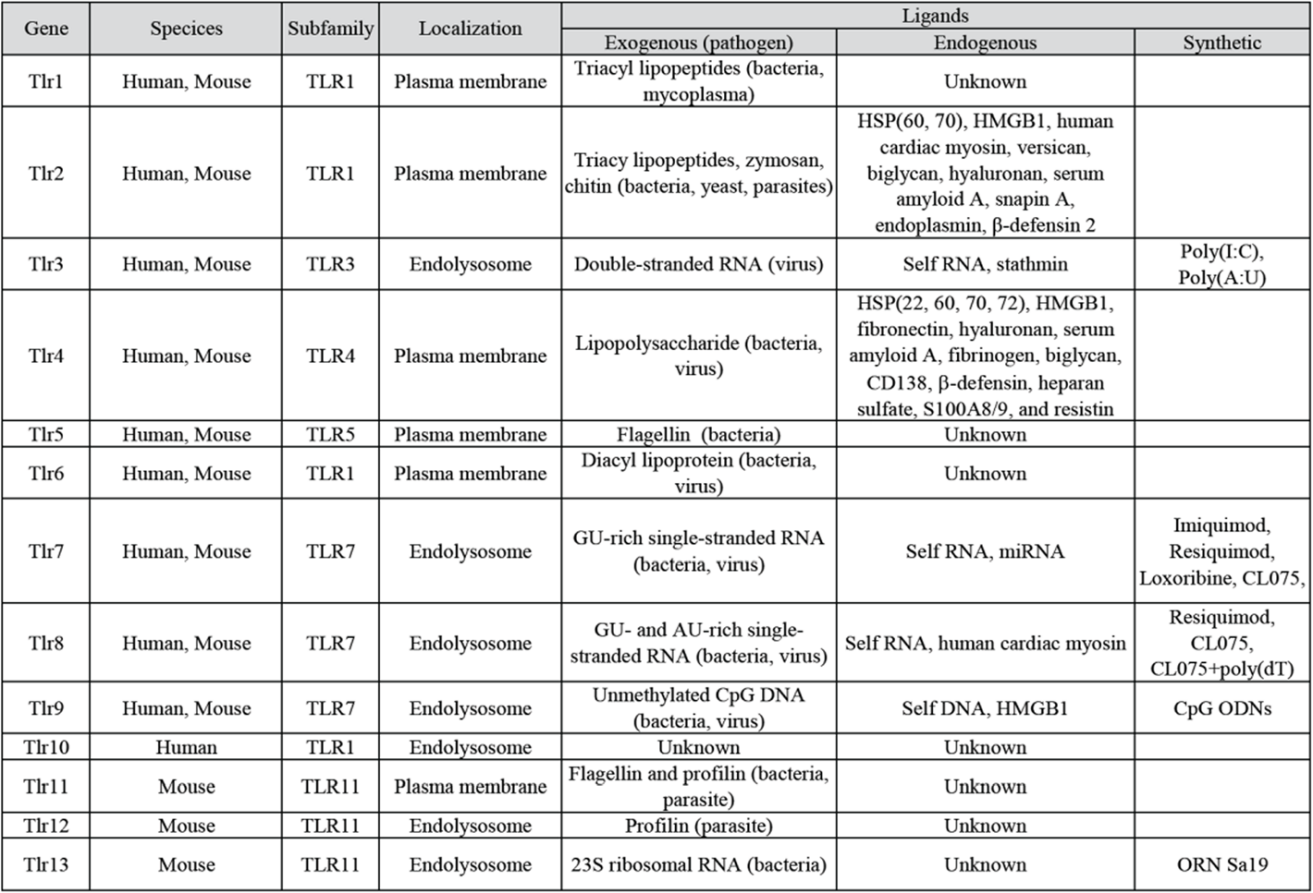
The TLR family in mammals

Thus far, ten TLRs have been identified in humans and twelve in mice. Both humans and mice express TLR1–9. Humans but not mice express TLR10, whereas mice have TLR11, TLR12, and TLR13 that are lacking in humans (Table 1). Based on sequence similarities, TLR1–13 can be grouped into six subfamilies, i.e., the TLR1, TLR3, TLR4, TLR5, TLR7 and TLR11 subfamilies (Table 1) [35, 36]. Closely-related TLRs recognize similar microbial molecules. For example, TLR7 and TLR8 both recognize single-strand RNA (ssRNA) [37, 38]. In addition, members of the same subfamily tend to form heterodimers to detect their ligands. For instance, TLR2 forms dimer with TLR1 or TLR6 to recognize a wide range of PAMPs, and TLR11-TLR12 dimer may bind to profilin to trigger a response against *Toxoplasma gondii* [39]. TLRs can also be divided into two groups based on their subcellular localization: (1) TLR1, TLR2, TLR4, TLR5, TLR6, TLR10 and TLR11 on the plasma membrane; and (2) endosomal TLRs, including TLR3, TLR7, TLR8, TLR9, TLR12 and TLR13 (Table 1) [40].

### Classical signaling pathways of TLRs in innate immunity

**Fig. 2:**
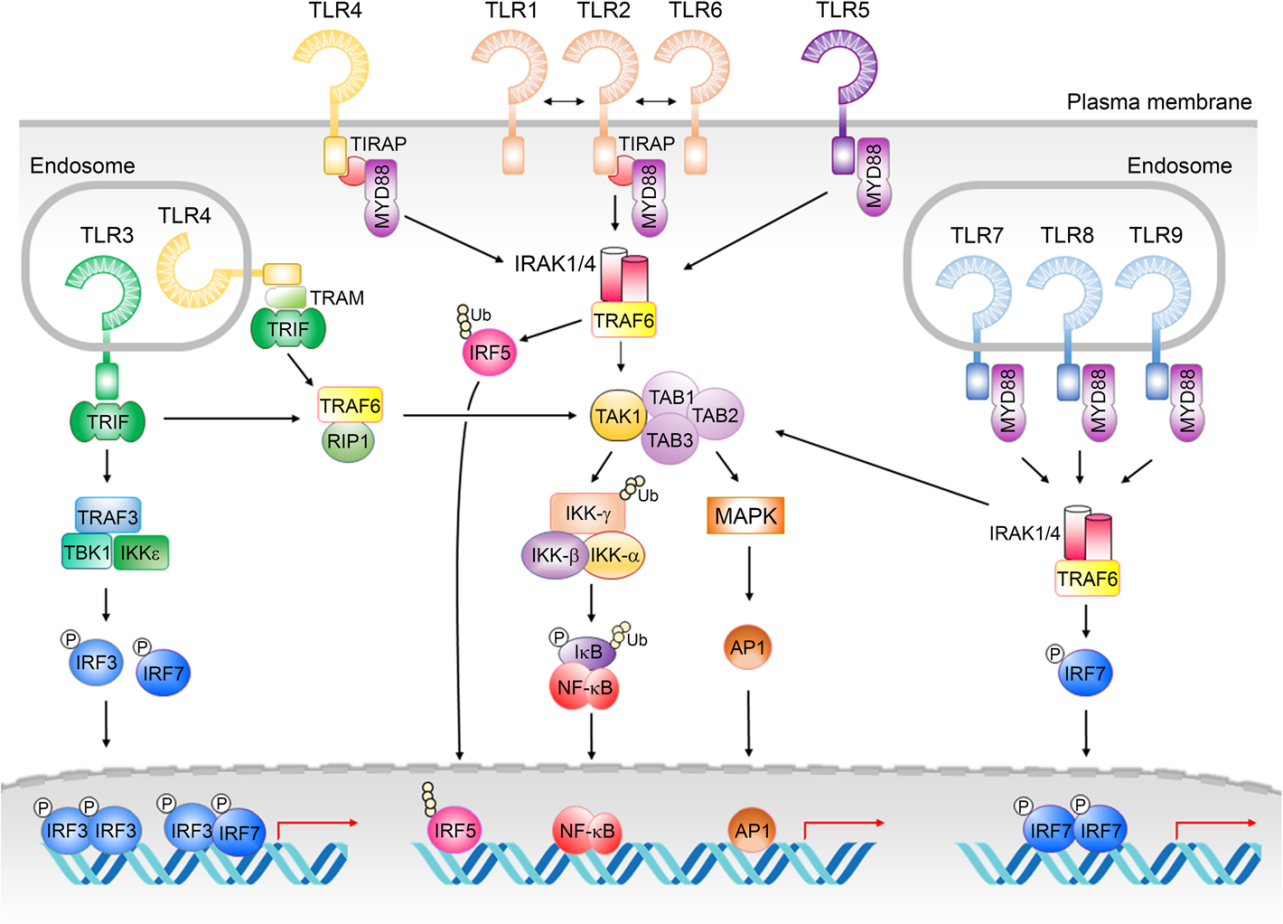
Classical TLR signaling pathways. MYD88 and TRIF are two major TIR domain-containing adaptors downstream of TLRs. IRFs, NF-κB and AP1 are three common downstream transcriptional factors in TLR pathways that regulate gene expression. Detailed descriptions are provided in the main text

Classical TLR signaling is mediated by five TIR domain-containing adaptors: myeloid differentiation primary response 88 (MYD88); TIR domain-containing adapter-inducing interferon-β (TRIF; also known as TICAM-1); TIR domain-containing adaptor protein (TIRAP); TRIF-related adaptor molecule (TRAM); and Sterile alpha and TIR motif-containing protein 1 (SARM1). TLR signaling is determined via interactions through the TIR domains of TLRs and their adaptors. Upon ligand binding, TLRs form homo- or hetero-dimers and transduce the signals to the MYD88- and TRIF-dependent pathways [41, 42]. MYD88 contains an N-terminal death domain, an intermediate domain, and a C-terminal TIR domain [43, 44], and it is the major adaptor protein for TLRs to trigger innate immune responses (Fig. 2). TRIF consists of an N-terminal globular helical domain, a TBK1-binding motif, TRAF6- and TRAF2-binding motifs, a TIR domain, and a C-terminal receptor-interacting protein homotypic interaction motif (RHIM) [45], and it mediates TLR3 and TLR4 signals (Fig. 2). MYD88 or TRIF is either recruited to the activated TLR directly, or indirectly through TIRAP and TRAM, to transduce the signal and subsequently induce expression of inflammatory cytokines and type I interferons [41, 42]. SARM1 is originally identified as a negative regulator of TRIF-dependent signaling that attenuates the innate immune response [46]. However, several studies suggest that SARM1 is predominantly expressed in neurons but not peripheral tissues, and that it plays important roles in regulating neuronal morphology [47], neural activity [48], autism-like behaviors [49] and wallerian degeneration [50–53]. More details about the function of SARM1 are available in previous reviews [5, 54].

### Neuronal TLRs modulate neurogenesis and neuronal differentiation

The innate immune and nervous systems emerged concurrently during evolution and before the mesoderm and adaptive immunity arose. *Hydra* (Phylum Cnidaria) is a simple organism with only ectoderm and endoderm, and it also expresses TLRs to recognize microbes [55]. It has been suggested that its simple nervous system can control the resident population of beneficial microbes, and that microbes affect *Hydra* behaviors by directly interfering with neuronal function [55]. Although direct evidence is still lacking, it seems possible that Hydra neurons use TLRs to directly recognize microbes and to consequently alter neuronal activity. In higher eukaryotic animals, particularly vertebrates, brains are considered an immune-privileged organ. The blood-brain barrier is largely responsible for isolating brain cells from peripheral immune cells and exogenous pathogens. There is a resident population of phagocytic cells in brain, i.e. microglia, that effect local inflammatory responses in the brain. Like microglia, neurons have been found to express several TLRs [56], though their expression levels are much lower than for microglia or macrophages [57, 58]. The low expression levels of TLRs in neurons are consistent with the fact that neurons are not responsible for triggering global innate immune responses. Thus, the question remains as to why neurons retain the ability to express TLRs in an immune-privileged environment. Is there any physiological reason to maintain TLR expression in neurons?

In rodents, TLRs have been reported to regulate neuronal progenitor cell (NPC) proliferation and neuronal morphology (including axon and dendrite outgrowth, and synapse formation) in mammalian brains even in the absence of infectious agents or tissue damage [5, 54, 59–61]. During neurogenesis, TLR2, TLR3 and TLR4 are present in NPCs and have distinct effects on NPC proliferation [28, 62]. For example, TLR3 negatively regulates embryonic NPC proliferation [63]. In TLR3-deficient mice, hippocampal CA1 and dentate gyrus volumes are increased and adult hippocampal neurogenesis is enhanced [64]. TLR2 and TLR4 are also expressed in adult NPCs [59]. Loss of TLR2 impairs hippocampal neurogenesis, whereas TLR4 deficiency upregulates neuronal proliferation and differentiation [59].

The functions and signaling pathways of TLR3, TLR7, and TLR8 in neuronal differentiation have been well investigated using both in vivo and in vitro knockdown systems and several knockout mouse lines [54, 60, 61, 65, 66]. These studies have demonstrated that specifically reducing expression or activation of TLR3, TLR7, or TLR8 in neurons results in abnormal neuronal differentiation and maturation in a cell-autonomous manner [54, 60, 61].

Both TLR7 and TLR8 detect single-stranded RNA (ssRNA) in endosomes, whereas TLR3 recognizes double-stranded RNA (dsRNA) and the synthetic ligand poly(I:C). Axonal growth is negatively controlled by TLR7 and TLR3, but not by TLR8 [54, 60, 61, 66]. Moreover, activations of TLR3, TLR7 and TLR8 all downregulate dendritic outgrowth [54, 60, 61]. While *Tlr7* RNA levels are almost constant, *Tlr8* RNA levels are increased as neuronal cultures mature [54]. Knockdown of *Tlr7* and *Tlr8* in vivo has different temporal effects on dendritic morphogenesis. Upon *Tlr7* knockdown beginning at embryonic day 15.5, layer 2/3 cortical neurons exhibited more complex dendritic arborization at postnatal day (P) 7 and P14, but not at P21 [60]. Conversely, *Tlr8* knockdown had an effect on dendritic arborization at P14 and P21, but not at P7 [54]. These distinct temporal effects are likely relevant to the expression timing of *Tlr7* and *Tlr8* and/or the presence of their endogenous ligands [54, 60].

TLR3, TLR7 and TLR8 carry out different functions during dendritic spine formation. TLR3 activation at P4 and P5 results in higher density but smaller dendritic spines of cortical layer 5 neurons at P21 [61]; a phenotype reminiscent of some autism spectrum disorders (ASD) [67–69]. Activation of TLR8 in cultured cortical neurons also increases dendritic spine density, but spine size seems unaffected [54], whereas TLR7 activation has no effect on dendritic spine morphogenesis (our unpublished data). Interestingly, though spine density is increased upon TLR8 activation, miniature excitatory synaptic currents (mEPSCs) are not changed at all [54]. Thus, the increased spine density likely compensates for the shorter dendrites caused by TLR8 activation.

In conclusion, the evidence indicates that TLRs differentially control neurogenesis and neuronal differentiation.

### TLRs regulate synaptic physiology and mouse behaviors

Apart from neuronal morphology, gain or loss of function of TLRs also affects synaptic plasticity and mouse behaviors. In cultured hippocampal neurons, TLR3 activation by poly(I:C) reduced spontaneous action potential firing via reducing sodium current in a TRIF-dependent manner [70]. The treatment also reduces surface expression of AMPAR and results in lower frequency and amplitude of mEPSCs [70]. When poly(I:C) was applied in vivo at embryonic day 15 and 17, long-term potentiation (LTP) was impaired at postnatal days 28–31 [71]. However, acute intraperitoneal administration of poly(I:C) for 4 h did not alter LTP of hippocampus [72]. Thus, embryonic treatment of poly(I:C) likely alters neurodevelopment and therefore influences synaptic plasticity. For TLR4, LPS treatment at postnatal stage activates astrocytes and thus promotes synaptogenesis and alters frequency and amplitude of mEPSC in hippocampal CA1 neurons [73].

The change of synaptic activity upon TLRs activation may echo the effect of TLRs on mouse behaviors. For example, systematic challenge of prenatal or neonatal mice with lipopolysaccharide (LPS; a TLR4 agonist) or poly(I:C) (a TLR3 agonist) causes autism-like (e.g. impaired social interaction) and schizophrenic-like behaviors, and increases anxiety [31, 74, 75]. Recently, prenatal immune challenge in mice has been shown to result in several sex-dependent behavioral deficits. For instance, maternal immune activation via poly(I:C) to trigger TLR3 activation induced anxiety-like and schizophrenia-like behaviors in male but not female mice [76]. Prenatal TLR7 activation by administration of imiquimod also results in sex-biased differences in anxiety-like behavior, repetitive behavior (self-grooming and marble burying), and disrupted social behavior [77]. These neuropsychiatric symptoms are due to improper brain development [31, 78].

In addition, deletion of *Tlr* genes also exhibit abnormal behavioral phenotypes that are highly related to dysfunction of the central nervous system (CNS). For example, *Tlr2* knockout mice develop schizophrenia-like behaviors and age-related obesity [79, 80]. *Tlr3* null mice present enhanced spatial learning and memory, but also exhibit anxiety and amygdala-dependent fear memory defects [64]. *Tlr4* deficiency in mice enhances motor functions and spatial memory acquisition and memory retention [81]. Interestingly, blockage of TLR4 signaling by infusing a TLR4 antagonist into the cerebral ventricles of adult mice does not affect cognitive behavior but induces anxiety [81]. This outcome suggests that the cognitive defects of *Tlr4* mutant mice are due to abnormal neuronal circuit formation during development. Furthermore, lack of *Tlr7* leads to less anxiety and aggression, better olfaction, and worse contextual fear memory in adult mice [82]. Moreover, 2-week-old *Tlr7* knockout mice exhibit lower exploratory activity [60]. These abnormal behaviors of *Tlr7* knockout mice may be linked to dysregulation of neuronal morphogenesis during the first two postnatal weeks [60]. Currently, synaptic physiology of CNS has been only investigated in *Tlr4* and *Tlr7* deficient mice. *Tlr4* mutant mice showed the impairment of long-term depression (LTD) but not LTP in nucleus accumbens [83] and *Tlr7* knockout mice exhibited LTP defect in hippocampus [82]. It would be worthy to investigate the synaptic activity in different brain regions of these *Tlr* mutant mice to further correlate the distinct behavior outcomes.

Since neuronal TLRs regulate neuronal morphology and activity, they likely contribute at least partially to the effects of TLR deletion or activation on mouse behaviors.

### Long-lasting effects of TLR activation

Innate immunity is also known to exhibit long-lasting memory via epigenetic reprogramming in innate immune cells. So-called “innate immune memory” is achieved by histone modification, DNA methylation and/or regulation by miRNAs and long-nonconding RNAs [84–86]. As summarized above, TLR activation at prenatal and neonatal stages results in abnormal dendritic spines and behavioral alteration in adults. This outcome suggests that TLR activation has a long-lasting effect on neural function. It is likely that neurons also exhibit a mechanism similar to innate immune memory to epigenetically control gene expression and thereby have a long-lasting effect on neuronal morphology and function. Indeed, prenatal immune activation by maternal treatment with poly(I:C) changes the genome-wide landscape of DNA methylation in the brains of adult offspring. Both hyper- and hypo-methylated CpG have been identified at many distinct loci, including genes involved in interneuron differentiation, the Wnt pathway and neuronal development [87, 88]. However, it is still unclear whether and how poly(I:C) treatment results in epigenetic reprogramming of neurons. Bisulfite genomic sequencing of different brain cells (such as neurons, microglia and astrocytes) may be applied to address this issue.

### Non-classical TLR signaling

**Fig. 3:**
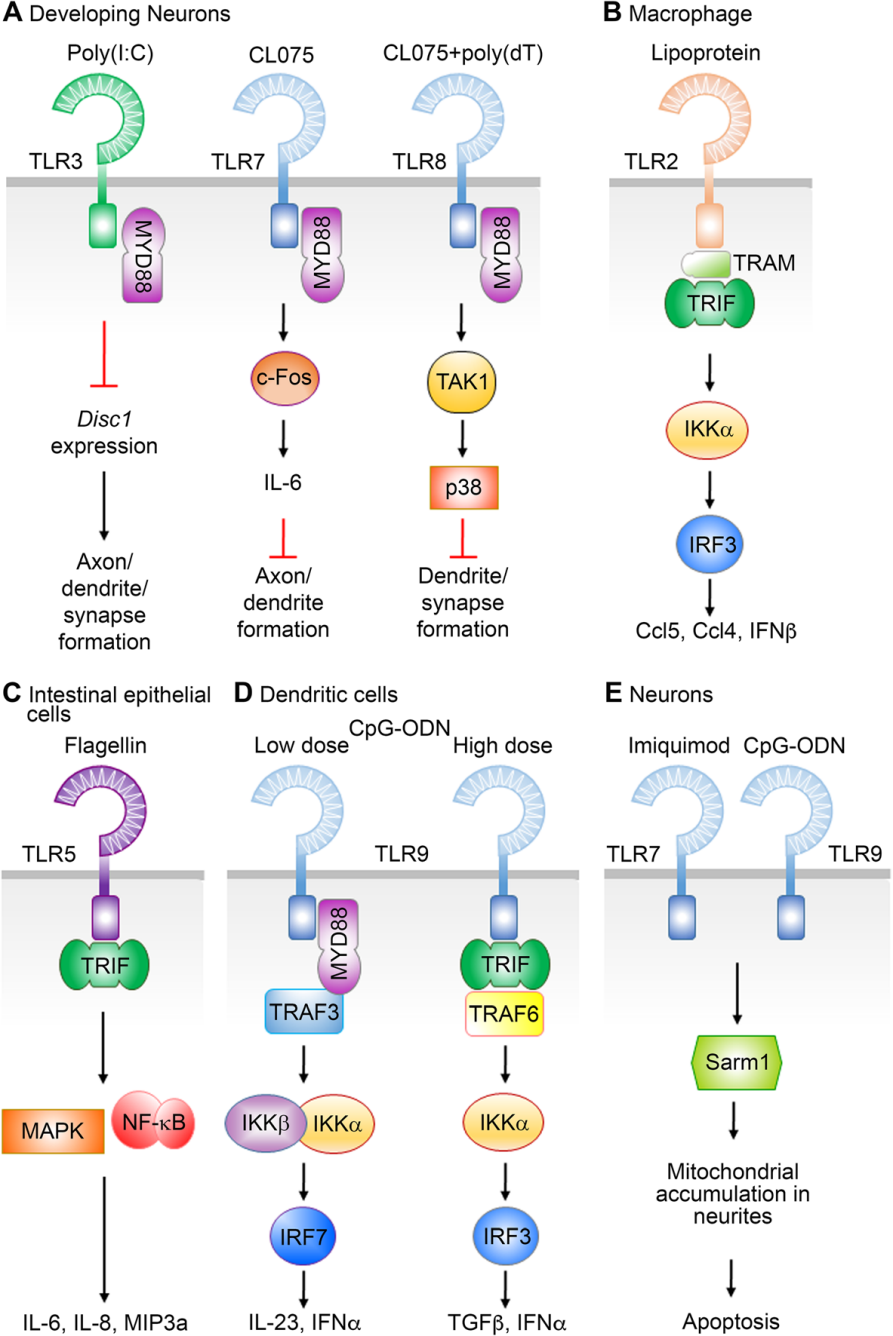
Non-classical TLR signaling pathways. **a** Three different endosomal TLRs use MYD88 to activate different pathways to fine-tune neuronal morphology. **b** TLR2 activates downstream signaling via TRIF in macrophages. **c** TLR5 can also use TRIF to deliver signal. **d** Different concentrations of ligand activate different TLR9 pathways in dendritic cells. **e** In addition to MYD88, TLR7 and TLR9 also use SARM1 to trigger cell death of neurons

Several studies have shown that some TLRs use different approaches to transduce signaling (Fig. 3). For example, in addition to MYD88, TLR2 can also use TRIF and TRAM to induce expression of type I interferons and the chemokine *Ccl5* in macrophages [89, 90]. TRIF is able to mediate TLR5 signaling in intestinal epithelial cells under flagellin challenge [91]. TLR9 acts through TRIF, but not MYD88, to promote a tolerogenic response in plasmacytoid dendritic cells [92]. Moreover, TLR7/9 signaling has been shown to require Sarm1 to induce apoptosis in neurons [93].

MYD88 is required by TLR3, TLR7 and TLR8 to regulate neuronal morphology. Activated TLR7 recruits MYD88 to activate the c-FOS and IL-6 cascade, thereby restricting axon and dendrite outgrowth [60]. IL-6 is required for TLR7 to downregulate dendritic growth, since IL-6^−/−^ neurons are insensitive to TLR7 activation [60]. The action of TLR7 in neurons is similar to that of the classical TLR7 pathway in immune cells, but expression levels of IL-6 are much lower in neurons than in immune cells [60]. The very low expression levels of IL-6 may account for the cell-autonomous effect of TLR7 on neuronal morphology. In contrast to the signal pathway of TLR7 in neurons and the action of TLR8 in immune cells, TLR8 activation in neurons does not induce cytokine expression [54, 60, 94]. Transcriptomic profiling analysis has revealed that TLR8 activation induces p38 activation and ERK inhibition in cultured neurons [54]. Although MYD88 and TAK1 are downstream of TLR8, TLR8 shares very few downstream target genes with TLR7 based on RNA-seq results [54], suggesting distinct downstream pathways and effectors for TLR7 and TLR8 in neurons, even though both TLR7 and TLR8 recognize ssRNA.

For TLR3, it utilizes two distinct adaptor proteins to regulate two different biological events in neurons. As in non-neuronal cells, TLR3 activation acts via TRIF to trigger cytokine expression in neurons. However, TLR3 activation can also negatively regulate dendritic arborization and influence synapse formation via MYD88 [61]. TLR3-MYD88 signaling cell-autonomously controls neuronal morphology by downregulating *Disc1*, a gene highly relevant to neuropsychiatric disorders (Fig. 3a**)** [61]. Thus, although MYD88 is required for the function of these TLRs in neurons, the downstream pathways of MYD88 are obviously different among TLR3, TLR7 and TLR8 because only a very small proportion of the downstream regulated genes are shared among these three TLRs [54].

Together, these reports suggest that canonical TLR signal pathways may not be universally applied. Instead, the signal pathways and functions of TLRs can vary in distinct cell types and under various physiological conditions (Fig. 3).

### Operation of non-classical signaling pathways in neurons

In the field of signal transduction, one of the most challenging issues is to study how a specific receptor specifically activates diverse downstream signal pathways under different circumstances or in distinct cell types. As described above, various downstream signaling mechanisms exist in TLR pathways. Two possible mechanisms have been speculated on. One is direct vs. indirect interaction between TLR and MYD88 or TRIF. Most TLRs directly interact with MYD88, but TLR2 and TLR4 can interact indirectly with MYD88 through TIRAP [42, 95]. Similarly, TRIF can interact directly with TLR3 or indirectly associate with TLR4 via TRAM [96, 97]. The most striking feature of TLR signaling complexes is that the adaptor MYD88 forms a helical assembly to recruit the downstream kinases and activate the signaling cascade [98, 99]. Thus, it is reasonable to speculate that different combinations of adaptor molecules form distinct helical structures and recruit diverse downstream signaling molecules. The various signalosomes then lead to differential regulation of gene expression.

The second possibility is that different receptors may interact with adaptor proteins in distinct ways and thereby form diverse signalosomes. Evidence to support this possibility comes in the form of a study on the interaction between TLR3 and MYD88 [61]. Traditionally, MYD88 and TRIF use their TIR domains to interact with the TIR domains of TLRs. However, the TIR domain of MYD88 is not required for the interaction with the TLR3 TIR domain. Instead, the N-terminal Death domain and intermediate domain of MYD88 are involved in the interaction between TLR3 and MYD88 [61]. The N-terminal Death domain of MYD88 is known to mediate oligomerization for downstream signaling via the IRAK4-IRAK1/2 pathway [98]. The interaction between TLR3 and the N-terminal region of MYD88 possibly alters the binding partners of MYD88. Since the TIR domain of MYD88 is free when MYD88 binds TLR3, this domain may be able to interact with other TIR domain-containing signaling molecules to form different signalosomes.

In addition to the two possible mechanisms mentioned above, another possibility is divergent spatiotemporal expression of TLRs and TIR domain-containing adaptors at cellular and subcellular levels. This possibility is not mutually exclusive from the two previously described mechanisms, and may add a level of diversity to TLR signaling pathways. For different subcellular distribution, the well-studied example is TLR4. It delivers signal at either the plasma membrane or endosomes via different adaptors (MYD88 and TRIF, respectively) [100]. TLR3, TLR7 and TLR8 are expressed at endosomal compartments. However, it is unclear whether these endosomal TLRs exist at the same or distinct vesicles or cells. If they are actually localized at different types of vesicles or cells because of diverse microenvironments, they are very likely to form distinct signalosomes and trigger various signaling. To explore this possibility, super high-resolution microscopy with double or triple immunofluorescence staining using specific antibodies against different TLRs will be necessary.

The intestinal epithelium represents an excellent example of differential TLR expression in various cell types. In a recent study, five TLR reporter mice were used to monitor the expression of TLR2, TLR4, TLR5, TLR7 and TLR9 in the small intestine of mice. Reporter cassettes containing internal ribosome entrance sites and fluorescent proteins were fused to the 3’ ends of the TLR genes. Based on fluorescent protein signals, it was clear that TLR expression dramatically varied in the different cell types of the small intestine [101]. It would be intriguing to use those reporter mice to further examine TLR expression in other tissue types.

### TLR7 and TLR8 function differentially in neurons

**Fig. 4:**
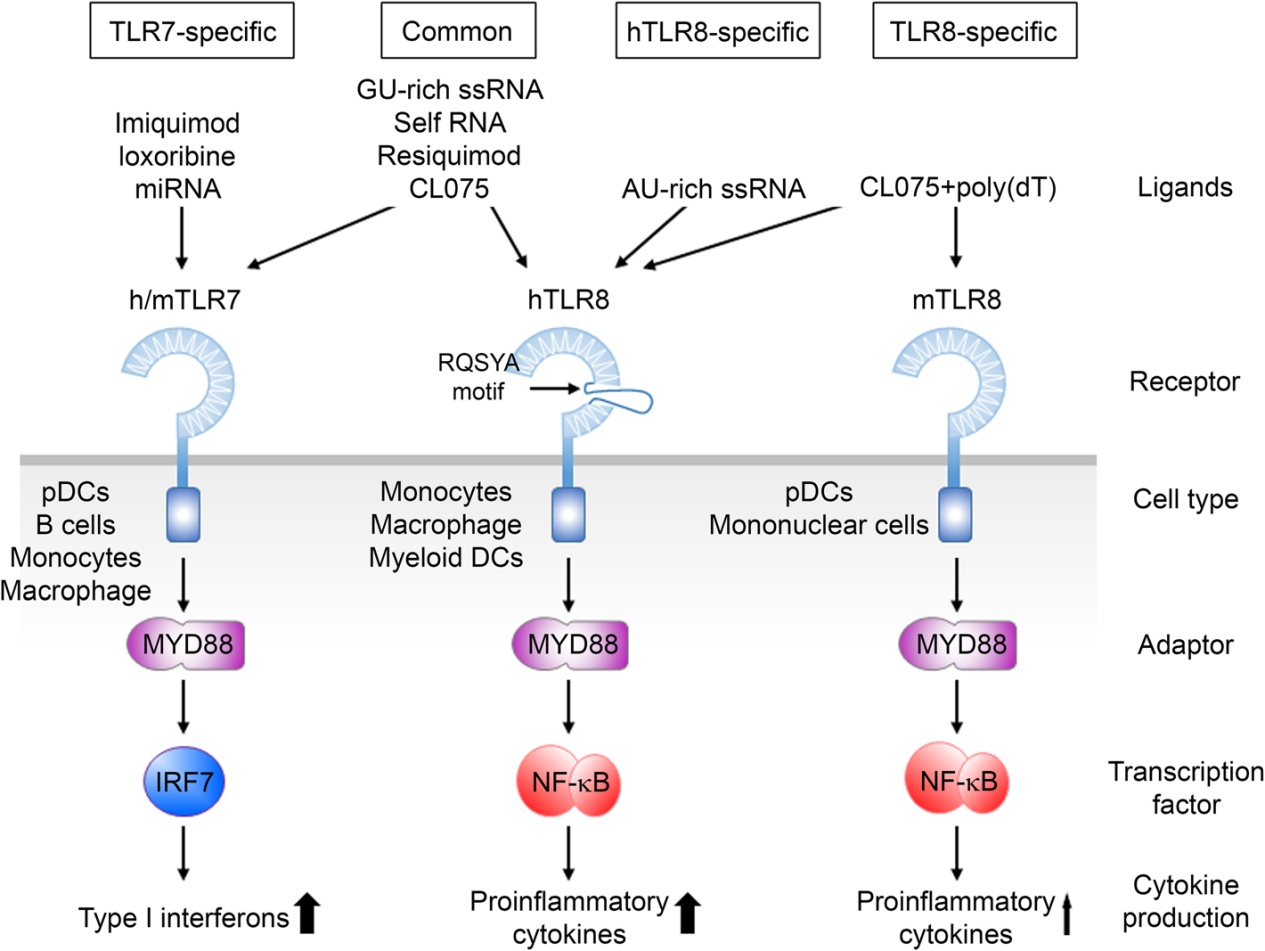
Comparison of TLR7 and TLR8 in terms of ligand binding, signaling pathways and their downstream effectors. Details are provided in the main text

In mammals, the *Tlr7* and *Tlr8* genes are located adjacently on the X chromosome. The TLR7 and TLR8 proteins exhibit highly similar amino acid sequences and homologous ligand recognition. However, differences between TLR7 and TLR8 in terms of ligand binding, signaling, and function have also been reported. First, for ligand binding, although both human TLR7 and TLR8 have been identified as sensors of viral or bacterial GU-rich ssRNA, resiquimod (R848), and CL075 [37, 38, 102, 103], they still exhibit ligand preferences (Fig. 4). TLR7, but not TLR8, can be activated by imiquimod (R837), loxoribine, and some miRNAs such as *Let7c* [10, 12]. TLR8 specifically reacts to stimulation by an AU-rich ssRNA and CL075 plus poly(dT) mixture, whereas TLR7 does not [54, 94, 102, 104]. Second, activation of TLR7 and TLR8 results in distinct cytokine production. Using specific agonists to trigger TLR7 or TLR8 activation and subsequent examination of the cytokine expression profile in several immune cells (such as monocytes, monocyte-derived dendritic cells (Mo-DCs), myeloid DCs, and plasmacytoid DCs (pDCs)) revealed that TLR7 activation predominantly induces IFNs and IFN-induced cytokine expression in pDCs, whereas TLR8 activation elicits high level of proinflammatory cytokines in monocytes, Mo-DCs, and myeloid DCs (Fig. 4) [102]. Such variation in TLR7 and TLR8 signaling could be due to divergent expression levels of these two receptors or to the presence of different downstream molecules in different cell types. These studies demonstrate that TLR7 and TLR8 have unique roles in regulating innate immune responses.

Notably, compared to human TLR8, murine TLR8 lacks the RQSYA motif in an undefined region immediately followed by LRR-14 [105]. By lacking this five amino-acid motif, murine TLR8 cannot respond to many human TLR8 ligands in the absence of poly(dT) [105]. Combination treatment of CL075 and poly(dT) triggers both human and mouse TLR8 activation and induces cytokine expression. However, the activity of mouse TLR8 is around 6-fold lower than that of human TLR8 [94, 105]. These findings suggest that rodent TLR8 is more selective for ligand binding and less efficient at inducing cytokine expression compared to human TLR8 (Fig. 4).

Despite the low cytokine production upon TLR8 activation, accumulating studies further support that mouse TLR8 is a functional and important receptor in many respects. As mentioned above, a recent paper showed that in vivo knockdown of mouse TLR8 in layer 2/3 cortical neurons increases dendritic arborization at P14 and P21 but not at P7, suggesting that mouse TLR8 is required to shape neuronal morphology and that its expression or its endogenous ligands are only present in mouse brain after the first postnatal week [54]. Another study reported that, as for human TLR8, murine TLR8 inhibits both murine and human TLR7 signaling [106]. suggesting that crosstalk exists between TLR7 and TLR8 signaling.

Furthermore, TLR7 has been associated with multiple sclerosis, an autoimmune disorder of the nervous system [107]. The balance of TLR7 and TLR8 expression seems to be critical for preventing autoimmune activation in peripheral tissues in mice, though the situation in neurons remains uncertain. Expression levels of *Tlr7* and *Tlr8* are negatively correlated with each other [108–113]. For instance, *Tlr7* expression is increased in *Tlr8* null mice and these mice exhibit lupus-like autoimmunity due to increased levels of RNP-specific autoantibodies [109, 110]. In neurons, *Tlr7* knockout increases *Tlr8* expression levels [54, 60]. Therefore, the evidence indicates that murine TLR7 and TLR8 possess unique functions in regulating autoimmunity and neuronal morphology, and although there is crosstalk between TLR7 and TLR8, they are not functionally redundant.

### TLRs fine-tune neuronal morphology

Genetic and functional studies have proven that TLRs are important for neuronal morphogenesis during development and that they can also influence animal behaviors. Generally, the brain is presumed to be a germ-free environment. The major source of TLR ligands in the brain is endogenous ligands such as miRNA, mRNA or DNA derived from exosomes and dead cells in the local environment. Since cytokine levels produced by neurons are much lower than those of non-neuronal cells [60, 114], neurons are not expected to efficiently trigger a global inflammatory response. Why have neurons evolved these complex systems to modulate their morphology?

**Fig. 5:**
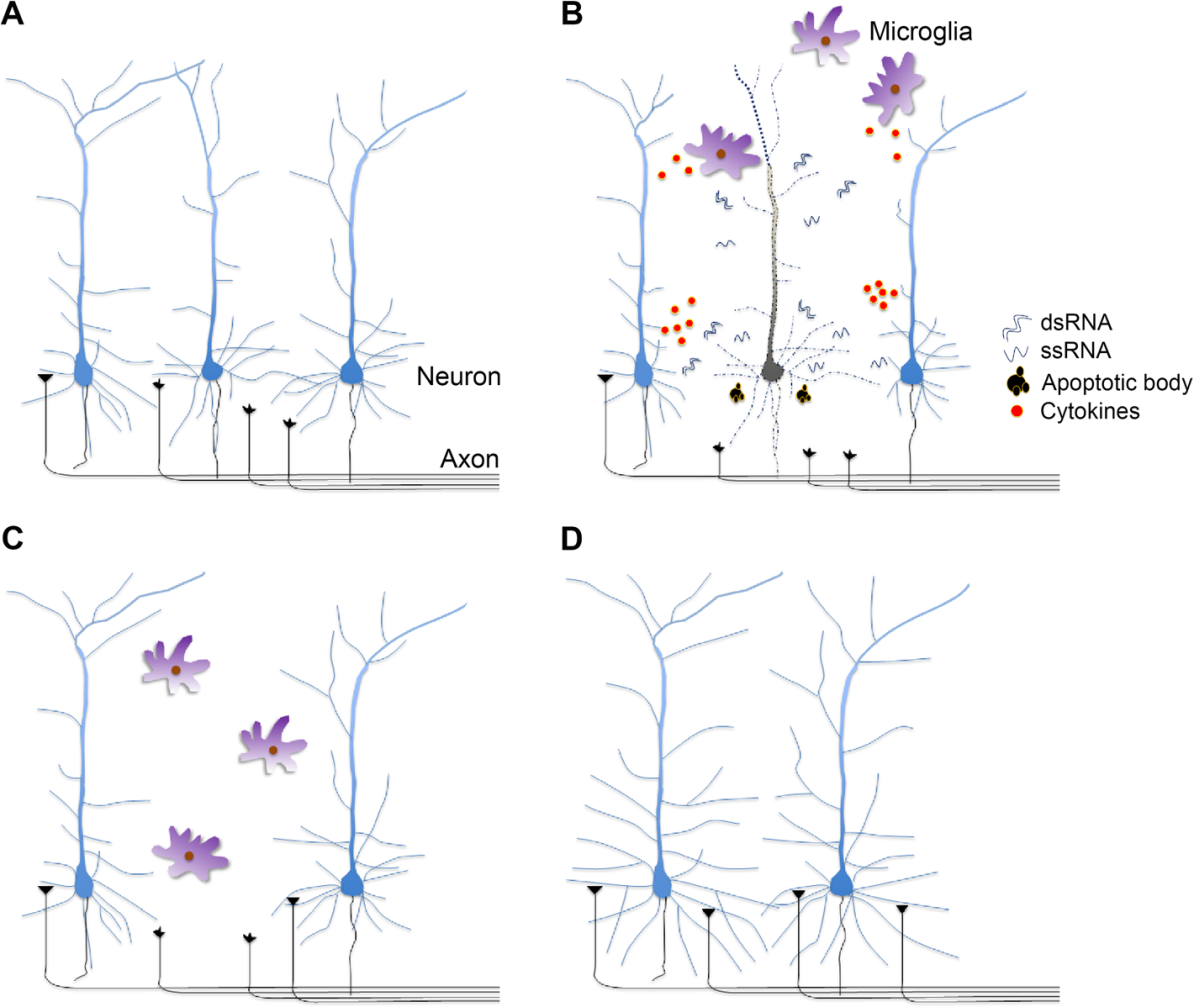
The action of neuronal TLRs during brain development. **a** During the first two postnatal weeks in the rodent brain, axon and dendrite outgrowth, as well as synapse formation, take place to establish connections and to build a functional brain. **b** Programmed cell death also occurs at the same time. DAMPs—including dsRNAs, ssRNAs, and apoptotic bodies—released from the dead neuron can activate TLRs in adjacent neurons. When those adjacent neurons receive the damage signals, they temporarily withdraw their dendrites to avoid growing into an unhealthy environment. The innervated axons from distal neurons likely also sense those damage factors and retract to prevent forming connections to a dead neuron. Meanwhile, activation of TLRs in neurons triggers local release of low levels of inflammatory cytokines and chemokines to attract nearby microglia. After entering the damaged zone, activated microglia engulf the remaining dead neuronal areas. **c** Once microglia have cleaned up the damaged area, adjacent neurons may regrow their dendrites. Axons projected from distal neurons may also resume extension to find their targets. **d** Later, the whole system is stabilized and the proper circuitry is established

In the first postnatal week, 90% of cells in rodent brains are neurons [115, 116], whereas the proportion of professional immune cells (microglia) is about 2–3% [117]. The proportion of microglia increases to 7–8% in the second postnatal week, and reaches ~ 10% in adult mouse brains [117]. During the first two postnatal weeks in rodent brains, neurons are most active at extending their dendrites and axons and forming connections. Programmed cell death of neurons also peaks concurrently [116]. Since microglial abundance remains low and may be less efficient at protecting entire brain regions by removing dead cells during this period [116, 118], neurons are more likely to encounter DAMPs derived from nearby dying cells. Therefore, we hypothesize that the purpose of TLR activation in neurons during brain development is to establish an alarm system that facilitates proper development of neuronal circuits (Fig. 5). During axonal and dendrite outgrowth and synapse formation in the first two postnatal weeks, neurons sense DNA or RNA from dead cells and then activate the nucleic acid-sensing TLRs. Consequently, neurons withdraw their dendrites and/or axons. Meanwhile, activation of TLR3 and TLR7 results in neuronal expression of cytokines. Although the levels are too low to induce global inflammatory responses, they might be sufficient to recruit nearby microglia to the damaged site for recovery (Fig. 5b). These actions ensure that neurons do not grow into unhealthy environments and facilitate correct connections between healthy neurons. Furthermore, a benefit of the very low level of inflammatory cytokines produced by neurons is to trigger localized immune activation instead of inducing a global inflammation storm in the developing brain. Through these complex and elegant mechanisms, neurons effectively detect changes in their environment during development and can fine-tune their morphology accordingly to establish the appropriate circuitry (Fig. 5).

A potential approach to further elucidating the role of neuronal TLR-mediated regulation is cell type-specific deletion of *Tlr* genes. Neuron- or microglia-specific knockout mice will be powerful tools for further studies of this topic. Furthermore, localized injury at early developmental stages combined with live imaging will aid investigations of whether neurons employ their own TLRs to detect proximal danger or damage signals to fine-tune their morphology.

## Conclusions and outlook

As ancient pattern recognition receptors [119], Toll/TLRs have evolved to sense diverse environmental cues and developmental signals that trigger the innate immune response and various cellular responses. In this review, the cited studies on neuronal TLR3, TLR7 and TLR8 reveal several interesting and important points about the physiological functions and regulation of TLRs in mammals. Those are concluded as follows.

First of all, the combined studies on neurons and other cell types clearly demonstrate that different TLRs may use distinct adaptors to activate different signal pathways. Downstream pathways can vary in different cell types even for the same receptor. Thus, in exploring the functions and/or signal pathways of a specific TLR in a specific type of cell, all possibilities must be considered. Importantly, the causal relationship between an identified pathway and a specific function needs to be proven. For instance, TLR3 uses two distinct adaptors to control different neuronal responses, i.e. TRIF for cytokine expression and MYD88 for altering neuronal morphology [61]. These two distinct pathways and the corresponding effects in neurons were validated by causal relationship experiments. Since the array of signalosomes for different TLRs in different cell types are likely the critical factors determining the diverse functions of TLRs, it would be very informative to reveal the structural features of different TLRs in combination with different TIR-domain adaptors, including MYD88, TRIF, TIRAP, TRAM and even SARM1. Such structural study would likely explain how different signal pathways are possible.

Second, TLR functions are not always mediated by cytokines. For example, TLR8 activation in neurons does not induce expression of cytokines and chemokines [54]. Although cytokines can be induced by TLR3 activation in neurons, cytokines are not required for TLR3 to downregulate neuronal morphology [61]. Thus, cytokine production can only represent one possible indicator of TLR activation. To control neuronal morphology, the regulators of actin and microtubule cytoskeletons are potential downstream effectors of TLRs, since the expression levels of Rho family pathway is altered in leukocytes upon TLR2 activation [120]. More investigations in neurons are required to address the possibility.

Third, neurons use TLRs to sense both exogenous and intrinsic danger signals to fine-tune their cellular structures and consequently alter neuronal connectivity. The main purpose of neuronal TLR activation is not to induce a global inflammatory response. It is possible that TLRs in other types of cells also carry out other functions distinct from that with respect to inflammation. It will be interesting to investigate this possibility.

Fourth, since innate immune responses have been associated with a variety of neurological diseases, including neuropsychiatric disorders and neurodegenerative diseases, and apart from the professional innate immune responses mediated by microglia and the peripheral immune system, the innate immune machinery in neurons provides another level of regulation that renders more complex the neuroinflammatory response.

Finally, recently developed TLR reporter mice provide the possibility of systematically examining TLR expression at the cellular and subcellular levels [101]. They circumvent the need for reliable TLR antibodies, which is important given that the specificity of the current crop of commercially available TLR antibodies has always been a concern. These reporter mice will be powerful tools in further establishing the action of TLRs in mammals and will hopefully help reveal new insights into the functions of TLRs beyond immunity.

## References

[1] Warrington R, Watson W, Kim HL, Antonetti FR (2011). An introduction to immunology and immunopathology. Allergy Asthma Clin Immunol.

[2] Dempsey PW, Vaidya SA, Cheng G (2003). The art of war: innate and adaptive immune responses. Cell Mol Life Sci.

[3] Brubaker SW, Bonham KS, Zanoni I, Kagan JC (2015). Innate immune pattern recognition: a cell biological perspective. Annu Rev Immunol.

[4] Takeuchi O, Akira S (2010). Pattern recognition receptors and inflammation. Cell.

[5] Liu HY, Chen CY, Hsueh YP (2014). Innate immune responses regulate morphogenesis and degeneration: roles of toll-like receptors and Sarm1 in neurons. Neurosci Bull.

[6] Barrat FJ, Meeker T, Gregorio J, Chan JH, Uematsu S, Akira S, Chang B, Duramad O, Coffman RL (2005). Nucleic acids of mammalian origin can act as endogenous ligands for toll-like receptors and may promote systemic lupus erythematosus. J Exp Med.

[7] Dellacasagrande J (2009). Ligands, cell-based models, and readouts required for toll-like receptor action. Methods Mol Biol.

[8] Czirr E, Wyss-Coray T (2012). The immunology of neurodegeneration. J Clin Invest.

[9] Kondo T, Kawai T, Akira S (2012). Dissecting negative regulation of toll-like receptor signaling. Trends Immunol.

[10] Lehmann SM, Kruger C, Park B, Derkow K, Rosenberger K, Baumgart J, Trimbuch T, Eom G, Hinz M, Kaul D, Habbel P, Kalin R, Franzoni E, Rybak A, Nguyen D, Veh R, Ninnemann O, Peters O, Nitsch R, Heppner FL, Golenbock D, Schott E, Ploegh HL, Wulczyn FG, Lehnardt S (2012). An unconventional role for miRNA: let-7 activates toll-like receptor 7 and causes neurodegeneration. Nat Neurosci.

[11] Park CK, Xu ZZ, Berta T, Han Q, Chen G, Liu XJ, Ji RR (2014). Extracellular microRNAs activate nociceptor neurons to elicit pain via TLR7 and TRPA1. Neuron.

[12] Liu HY, Huang CM, Hung YF, Hsueh YP (2015). The microRNAs Let7c and miR21 are recognized by neuronal toll-like receptor 7 to restrict dendritic growth of neurons. Exp Neurol.

[13] Man SM, Karki R, Kanneganti TD (2016). AIM2 inflammasome in infection, cancer, and autoimmunity: role in DNA sensing, inflammation, and innate immunity. Eur J Immunol.

[14] Monteith AJ, Kang S, Scott E, Hillman K, Rajfur Z, Jacobson K, Costello MJ, Vilen BJ (2016). Defects in lysosomal maturation facilitate the activation of innate sensors in systemic lupus erythematosus. Proc Natl Acad Sci U S A.

[15] Lian Q, Xu J, Yan S, Huang M, Ding H, Sun X, Bi A, Ding J, Sun B, Geng M (2017). Chemotherapy-induced intestinal inflammatory responses are mediated by exosome secretion of double-strand DNA via AIM2 inflammasome activation. Cell Res.

[16] Anderson KV, Jurgens G, Nusslein-Volhard C (1985). Establishment of dorsal-ventral polarity in the Drosophila embryo: genetic studies on the role of the toll gene product. Cell.

[17] Hashimoto C, Hudson KL, Anderson KV (1988). The toll gene of Drosophila, required for dorsal-ventral embryonic polarity, appears to encode a transmembrane protein. Cell.

[18] Williams MJ, Rodriguez A, Kimbrell DA, Eldon ED (1997). The 18-wheeler mutation reveals complex antibacterial gene regulation in Drosophila host defense. EMBO J.

[19] Ligoxygakis P, Bulet P, Reichhart JM (2002). Critical evaluation of the role of the toll-like receptor 18-wheeler in the host defense of Drosophila. EMBO Rep.

[20] Yagi Y, Nishida Y, Ip YT (2010). Functional analysis of toll-related genes in Drosophila. Develop Growth Differ.

[21] Foldi I, Anthoney N, Harrison N, Gangloff M, Verstak B, Nallasivan MP, AlAhmed S, Zhu B, Phizacklea M, Losada-Perez M, Moreira M, Gay NJ, Hidalgo A (2017). Three-tier regulation of cell number plasticity by neurotrophins and tolls in Drosophila. J Cell Biol.

[22] Anthoney N, Foldi I, Hidalgo A. Toll and Toll-like receptor signalling in development. Development. 2018:145(9). 10.1242/dev.156018 29695493

[23] Ward A, Hong W, Favaloro V, Luo L (2015). Toll receptors instruct axon and dendrite targeting and participate in synaptic partner matching in a Drosophila olfactory circuit. Neuron.

[24] Liu B, Zheng Y, Yin F, Yu J, Silverman N, Pan D (2016). Toll receptor-mediated hippo signaling controls innate immunity in Drosophila. Cell.

[25] Alpar L, Bergantinos C, Johnston LA (2018). Spatially restricted regulation of Spatzle/toll signaling during cell competition. Dev Cell.

[26] Germani F, Hain D, Sternlicht D, Moreno E, Basler K. The toll pathway inhibits tissue growth and regulates cell fitness in an infection-dependent manner. Elife. 2018;7. 10.7554/eLife.39939. 10.7554/eLife.39939 PMC6279345 30451683

[27] Katsukawa M, Ohsawa S, Zhang L, Yan Y, Igaki T (2018). Serpin facilitates tumor-suppressive cell competition by blocking toll-mediated Yki activation in Drosophila. Curr Biol.

[28] Barak B, Feldman N, Okun E (2014). Toll-like receptors as developmental tools that regulate neurogenesis during development: an update. Front Neurosci.

[29] Al-Haddad BJS, Jacobsson B, Chabra S, Modzelewska D, Olson EM, Bernier R, Enquobahrie DA, Hagberg H, Ostling S, Rajagopal L, Adams Waldorf KM, Sengpiel V (2019). Long-term Risk of Neuropsychiatric Disease After Exposure to Infection In Utero. JAMA Psychiatry.

[30] Gumusoglu SB, Stevens HE (2019). Maternal inflammation and neurodevelopmental programming: a review of preclinical outcomes and implications for translational psychiatry. Biol Psychiatry.

[31] Knuesel I, Chicha L, Britschgi M, Schobel SA, Bodmer M, Hellings JA, Toovey S, Prinssen EP (2014). Maternal immune activation and abnormal brain development across CNS disorders. Nat Rev Neurol.

[32] Choi GB, Yim YS, Wong H, Kim S, Kim H, Kim SV, Hoeffer CA, Littman DR, Huh JR (2016). The maternal interleukin-17a pathway in mice promotes autism-like phenotypes in offspring. Science.

[33] Bell JK, Mullen GE, Leifer CA, Mazzoni A, Davies DR, Segal DM (2003). Leucine-rich repeats and pathogen recognition in toll-like receptors. Trends Immunol.

[34] Gay NJ, Gangloff M (2007). Structure and function of toll receptors and their ligands. Annu Rev Biochem.

[35] Roach JC, Glusman G, Rowen L, Kaur A, Purcell MK, Smith KD, Hood LE, Aderem A (2005). The evolution of vertebrate toll-like receptors. Proc Natl Acad Sci U S A.

[36] Wang J, Zhang Z, Liu J, Zhao J, Yin D (2016). Ectodomain architecture affects sequence and functional evolution of vertebrate toll-like receptors. Sci Rep.

[37] Diebold SS, Kaisho T, Hemmi H, Akira S (2004). Reis e Sousa C. innate antiviral responses by means of TLR7-mediated recognition of single-stranded RNA. Science.

[38] Heil F, Hemmi H, Hochrein H, Ampenberger F, Kirschning C, Akira S, Lipford G, Wagner H, Bauer S (2004). Species-specific recognition of single-stranded RNA via toll-like receptor 7 and 8. Science.

[39] Koblansky AA, Jankovic D, Oh H, Hieny S, Sungnak W, Mathur R, Hayden MS, Akira S, Sher A, Ghosh S (2013). Recognition of profilin by toll-like receptor 12 is critical for host resistance to toxoplasma gondii. Immunity.

[40] Leone M, Moreau R (2014). Leukocyte toll-like receptor 2-mitochondria axis in sepsis: unraveling immune response sophistication. Anesthesiology.

[41] Yamamoto M, Sato S, Hemmi H, Hoshino K, Kaisho T, Sanjo H, Takeuchi O, Sugiyama M, Okabe M, Takeda K, Akira S (2003). Role of adaptor TRIF in the MyD88-independent toll-like receptor signaling pathway. Science.

[42] Kawai T, Akira S (2010). The role of pattern-recognition receptors in innate immunity: update on toll-like receptors. Nat Immunol.

[43] Bonnert TP, Garka KE, Parnet P, Sonoda G, Testa JR, Sims JE (1997). The cloning and characterization of human MyD88: a member of an IL-1 receptor related family. FEBS Lett.

[44] Medzhitov R, Preston-Hurlburt P, Kopp E, Stadlen A, Chen C, Ghosh S, Janeway CA (1998). MyD88 is an adaptor protein in the hToll/IL-1 receptor family signaling pathways. Mol Cell.

[45] Oshiumi H, Matsumoto M, Funami K, Akazawa T, Seya T (2003). TICAM-1, an adaptor molecule that participates in toll-like receptor 3-mediated interferon-beta induction. Nat Immunol.

[46] Carty M, Goodbody R, Schroder M, Stack J, Moynagh PN, Bowie AG (2006). The human adaptor SARM negatively regulates adaptor protein TRIF-dependent toll-like receptor signaling. Nat Immunol.

[47] Chen CY, Lin CW, Chang CY, Jiang ST, Hsueh YP (2011). Sarm1, a negative regulator of innate immunity, interacts with syndecan-2 and regulates neuronal morphology. J Cell Biol.

[48] Lin CW, Chen CY, Cheng SJ, Hu HT, Hsueh YP (2014). Sarm1 deficiency impairs synaptic function and leads to behavioral deficits, which can be ameliorated by an mGluR allosteric modulator. Front Cell Neurosci.

[49] Lin CW, Hsueh YP (2014). Sarm1, a neuronal inflammatory regulator, controls social interaction, associative memory and cognitive flexibility in mice. Brain Behav Immun.

[50] Osterloh JM, Yang J, Rooney TM, Fox AN, Adalbert R, Powell EH, Sheehan AE, Avery MA, Hackett R, Logan MA, MacDonald JM, Ziegenfuss JS, Milde S, Hou YJ, Nathan C, Ding A, Brown RH, Conforti L, Coleman M, Tessier-Lavigne M, Zuchner S, Freeman MR (2012). dSarm/Sarm1 is required for activation of an injury-induced axon death pathway. Science.

[51] Gerdts J, Summers DW, Sasaki Y, DiAntonio A, Milbrandt J (2013). Sarm1-mediated axon degeneration requires both SAM and TIR interactions. J Neurosci.

[52] Gerdts J, Brace EJ, Sasaki Y, DiAntonio A, Milbrandt J (2015). SARM1 activation triggers axon degeneration locally via NAD(+) destruction. Science.

[53] Gerdts J, Summers DW, Milbrandt J, DiAntonio A (2016). Axon self-destruction: new links among SARM1, MAPKs, and NAD+ metabolism. Neuron.

[54] Hung YF, Chen CY, Shih YC, Liu HY, Huang CM, Hsueh YP (2018). Endosomal TLR3, TLR7, and TLR8 control neuronal morphology through different transcriptional programs. J Cell Biol.

[55] Klimovich AV, Bosch TCG (2018). Rethinking the role of the nervous system: lessons from the Hydra Holobiont. Bioessays.

[56] Hanke ML, Kielian T (2011). Toll-like receptors in health and disease in the brain: mechanisms and therapeutic potential. Clin Sci (Lond).

[57] Zhang Y, Chen K, Sloan SA, Bennett ML, Scholze AR, O'Keeffe S, Phatnani HP, Guarnieri P, Caneda C, Ruderisch N, Deng S, Liddelow SA, Zhang C, Daneman R, Maniatis T, Barres BA, Wu JQ (2014). An RNA-sequencing transcriptome and splicing database of glia, neurons, and vascular cells of the cerebral cortex. J Neurosci.

[58] Shmueli A, Shalit T, Okun E, Shohat-Ophir G (2018). The toll pathway in the central nervous system of flies and mammals. NeuroMolecular Med.

[59] Rolls A, Shechter R, London A, Ziv Y, Ronen A, Levy R, Schwartz M (2007). Toll-like receptors modulate adult hippocampal neurogenesis. Nat Cell Biol.

[60] Liu HY, Hong YF, Huang CM, Chen CY, Huang TN, Hsueh YP (2013). TLR7 negatively regulates dendrite outgrowth through the Myd88-c-Fos-IL-6 pathway. J Neurosci.

[61] Chen CY, Liu HY, Hsueh YP (2017). TLR3 downregulates expression of schizophrenia gene Disc1 via MYD88 to control neuronal morphology. EMBO Rep.

[62] Okun E, Griffioen KJ, Mattson MP (2011). Toll-like receptor signaling in neural plasticity and disease. Trends Neurosci.

[63] Lathia JD, Okun E, Tang SC, Griffioen K, Cheng A, Mughal MR, Laryea G, Selvaraj PK (2008). Ffrench-constant C, Magnus T, Arumugam TV, Mattson MP. Toll-like receptor 3 is a negative regulator of embryonic neural progenitor cell proliferation. J Neurosci.

[64] Okun E, Griffioen K, Barak B, Roberts NJ, Castro K, Pita MA, Cheng A, Mughal MR, Wan R, Ashery U, Mattson MP (2010). Toll-like receptor 3 inhibits memory retention and constrains adult hippocampal neurogenesis. Proc Natl Acad Sci U S A.

[65] Ma Y, Li J, Chiu I, Wang Y, Sloane JA, Lu J, Kosaras B, Sidman RL, Volpe JJ, Vartanian T (2006). Toll-like receptor 8 functions as a negative regulator of neurite outgrowth and inducer of neuronal apoptosis. J Cell Biol.

[66] Cameron JS, Alexopoulou L, Sloane JA, DiBernardo AB, Ma Y, Kosaras B, Flavell R, Strittmatter SM, Volpe J, Sidman R, Vartanian T (2007). Toll-like receptor 3 is a potent negative regulator of axonal growth in mammals. J Neurosci.

[67] Hutsler JJ, Zhang H (2010). Increased dendritic spine densities on cortical projection neurons in autism spectrum disorders. Brain Res.

[68] Tang G, Gudsnuk K, Kuo SH, Cotrina ML, Rosoklija G, Sosunov A, Sonders MS, Kanter E, Castagna C, Yamamoto A, Yue Z, Arancio O, Peterson BS, Champagne F, Dwork AJ, Goldman J, Sulzer D (2014). Loss of mTOR-dependent macroautophagy causes autistic-like synaptic pruning deficits. Neuron.

[69] Penzes P, Cahill ME, Jones KA, VanLeeuwen JE, Woolfrey KM (2011). Dendritic spine pathology in neuropsychiatric disorders. Nat Neurosci.

[70] Ritchie L, Tate R, Chamberlain LH, Robertson G, Zagnoni M, Sposito T, Wray S, Wright JA, Bryant CE, Gay NJ, Bushell TJ (2018). Toll-like receptor 3 activation impairs excitability and synaptic activity via TRIF signalling in immature rat and human neurons. Neuropharmacology.

[71] Oh-Nishi A, Obayashi S, Sugihara I, Minamimoto T, Suhara T (2010). Maternal immune activation by polyriboinosinic-polyribocytidilic acid injection produces synaptic dysfunction but not neuronal loss in the hippocampus of juvenile rat offspring. Brain Res.

[72] Costello DA, Lynch MA (2013). Toll-like receptor 3 activation modulates hippocampal network excitability, via glial production of interferon-beta. Hippocampus.

[73] Shen Y, Qin H, Chen J, Mou L, He Y, Yan Y, Zhou H, Lv Y, Chen Z, Wang J, Zhou YD (2016). Postnatal activation of TLR4 in astrocytes promotes excitatory synaptogenesis in hippocampal neurons. J Cell Biol.

[74] Khandaker GM, Cousins L, Deakin J, Lennox BR, Yolken R, Jones PB (2015). Inflammation and immunity in schizophrenia: implications for pathophysiology and treatment. Lancet Psychiatry.

[75] Bilbo SD, Block CL, Bolton JL, Hanamsagar R, Tran PK (2018). Beyond infection - Maternal immune activation by environmental factors, microglial development, and relevance for autism spectrum disorders. Exp Neurol.

[76] Hui CW, St-Pierre A, El Hajj H, Remy Y, Hebert SS, Luheshi GN, Srivastava LK, Tremblay ME (2018). Prenatal immune challenge in mice leads to partly sex-dependent behavioral, microglial, and molecular abnormalities associated with schizophrenia. Front Mol Neurosci.

[77] Missig G, Robbins JO, Mokler EL, McCullough KM, Bilbo SD, McDougle CJ, Carlezon WA Jr. Sex-dependent neurobiological features of prenatal immune activation via TLR7. Mol Psychiatry. 2019. 10.1038/s41380-018-0346-4 PMC7515834 30610201

[78] Phillips M, Pozzo-Miller L (2015). Dendritic spine dysgenesis in autism related disorders. Neurosci Lett.

[79] Shechter R, London A, Kuperman Y, Ronen A, Rolls A, Chen A, Schwartz M (2013). Hypothalamic neuronal toll-like receptor 2 protects against age-induced obesity. Sci Rep.

[80] Park SJ, Lee JY, Kim SJ, Choi SY, Yune TY, Ryu JH (2015). Toll-like receptor-2 deficiency induces schizophrenia-like behaviors in mice. Sci Rep.

[81] Okun E, Barak B, Saada-Madar R, Rothman SM, Griffioen KJ, Roberts N, Castro K, Mughal MR, Pita MA, Stranahan AM, Arumugam TV, Mattson MP (2012). Evidence for a developmental role for TLR4 in learning and memory. PLoS One.

[82] Hung YF, Chen CY, Li WC, Wang TF, Hsueh YP (2018). Tlr7 deletion alters expression profiles of genes related to neural function and regulates mouse behaviors and contextual memory. Brain Behav Immun.

[83] Kashima DT, Grueter BA (2017). Toll-like receptor 4 deficiency alters nucleus accumbens synaptic physiology and drug reward behavior. Proc Natl Acad Sci U S A.

[84] Netea MG, Latz E, Mills KH, O'Neill LA (2015). Innate immune memory: a paradigm shift in understanding host defense. Nat Immunol.

[85] Netea MG, Joosten LA, Latz E, Mills KH, Natoli G, Stunnenberg HG, O'Neill LA, Xavier RJ (2016). Trained immunity: A program of innate immune memory in health and disease. Science.

[86] Melillo D, Marino R, Italiani P, Boraschi D (2018). Innate immune memory in invertebrate metazoans: a critical appraisal. Front Immunol.

[87] Basil P, Li Q, Dempster EL, Mill J, Sham PC, Wong CC, McAlonan GM (2014). Prenatal maternal immune activation causes epigenetic differences in adolescent mouse brain. Transl Psychiatry.

[88] Richetto J, Massart R, Weber-Stadlbauer U, Szyf M, Riva MA, Meyer U (2017). Genome-wide DNA methylation changes in a mouse model of infection-mediated neurodevelopmental disorders. Biol Psychiatry.

[89] Petnicki-Ocwieja T, Chung E, Acosta DI, Ramos LT, Shin OS, Ghosh S, Kobzik L, Li X, Hu LT (2013). TRIF mediates toll-like receptor 2-dependent inflammatory responses to Borrelia burgdorferi. Infect Immun.

[90] Nilsen NJ, Vladimer GI, Stenvik J, Orning MP, Zeid-Kilani MV, Bugge M, Bergstroem B, Conlon J, Husebye H, Hise AG, Fitzgerald KA, Espevik T, Lien E (2015). A role for the adaptor proteins TRAM and TRIF in toll-like receptor 2 signaling. J Biol Chem.

[91] Choi YJ, Im E, Chung HK, Pothoulakis C, Rhee SH (2010). TRIF mediates toll-like receptor 5-induced signaling in intestinal epithelial cells. J Biol Chem.

[92] Volpi C, Fallarino F, Pallotta MT, Bianchi R, Vacca C, Belladonna ML, Orabona C, De Luca A, Boon L, Romani L, Grohmann U, Puccetti P (2013). High doses of CpG oligodeoxynucleotides stimulate a tolerogenic TLR9-TRIF pathway. Nat Commun.

[93] Mukherjee P, Winkler CW, Taylor KG, Woods TA, Nair V, Khan BA, Peterson KE (2015). SARM1, not MyD88, mediates TLR7/TLR9-induced apoptosis in neurons. J Immunol.

[94] Gorden KK, Qiu XX, Binsfeld CC, Vasilakos JP, Alkan SS (2006). Cutting edge: activation of murine TLR8 by a combination of imidazoquinoline immune response modifiers and polyT oligodeoxynucleotides. J Immunol.

[95] Horng T, Barton GM, Medzhitov R (2001). TIRAP: an adapter molecule in the toll signaling pathway. Nat Immunol.

[96] Yamamoto M, Sato S, Hemmi H, Uematsu S, Hoshino K, Kaisho T, Takeuchi O, Takeda K, Akira S (2003). TRAM is specifically involved in the toll-like receptor 4-mediated MyD88-independent signaling pathway. Nat Immunol.

[97] Yamamoto M, Sato S, Hemmi H, Sanjo H, Uematsu S, Kaisho T, Hoshino K, Takeuchi O, Kobayashi M, Fujita T, Takeda K, Akira S (2002). Essential role for TIRAP in activation of the signalling cascade shared by TLR2 and TLR4. Nature.

[98] Lin SC, Lo YC, Wu H (2010). Helical assembly in the MyD88-IRAK4-IRAK2 complex in TLR/IL-1R signalling. Nature.

[99] Guven-Maiorov E, Keskin O, Gursoy A, VanWaes C, Chen Z, Tsai CJ, Nussinov R (2015). The architecture of the TIR domain Signalosome in the toll-like Receptor-4 signaling pathway. Sci Rep.

[100] Funami K, Matsumoto M, Oshiumi H, Inagaki F, Seya T (2017). Functional interfaces between TICAM-2/TRAM and TICAM-1/TRIF in TLR4 signaling. Biochem Soc Trans.

[101] Price AE, Shamardani K, Lugo KA, Deguine J, Roberts AW, Lee BL, Barton GM (2018). A map of toll-like receptor expression in the intestinal epithelium reveals distinct spatial, cell type-specific, and temporal patterns. Immunity.

[102] Gorden KB, Gorski KS, Gibson SJ, Kedl RM, Kieper WC, Qiu X, Tomai MA, Alkan SS, Vasilakos JP (2005). Synthetic TLR agonists reveal functional differences between human TLR7 and TLR8. J Immunol.

[103] Gantier MP, Tong S, Behlke MA, Xu D, Phipps S, Foster PS, Williams BR (2008). TLR7 is involved in sequence-specific sensing of single-stranded RNAs in human macrophages. J Immunol.

[104] Forsbach A, Nemorin JG, Montino C, Muller C, Samulowitz U, Vicari AP, Jurk M, Mutwiri GK, Krieg AM, Lipford GB, Vollmer J (2008). Identification of RNA sequence motifs stimulating sequence-specific TLR8-dependent immune responses. J Immunol.

[105] Liu J, Xu C, Hsu LC, Luo Y, Xiang R, Chuang TH (2010). A five-amino-acid motif in the undefined region of the TLR8 ectodomain is required for species-specific ligand recognition. Mol Immunol.

[106] Wang J, Shao Y, Bennett TA, Shankar RA, Wightman PD, Reddy LG (2006). The functional effects of physical interactions among toll-like receptors 7, 8, and 9. J Biol Chem.

[107] Du S, Itoh N, Askarinam S, Hill H, Arnold AP, Voskuhl RR (2014). XY sex chromosome complement, compared with XX, in the CNS confers greater neurodegeneration during experimental autoimmune encephalomyelitis. Proc Natl Acad Sci U S A.

[108] Guiducci C, Gong M, Cepika AM, Xu Z, Tripodo C, Bennett L, Crain C, Quartier P, Cush JJ, Pascual V, Coffman RL, Barrat FJ (2013). RNA recognition by human TLR8 can lead to autoimmune inflammation. J Exp Med.

[109] Demaria O, Pagni PP, Traub S, de Gassart A, Branzk N, Murphy AJ, Valenzuela DM, Yancopoulos GD, Flavell RA, Alexopoulou L (2010). TLR8 deficiency leads to autoimmunity in mice. J Clin Invest.

[110] Tran NL, Manzin-Lorenzi C, Santiago-Raber ML (2015). Toll-like receptor 8 deletion accelerates autoimmunity in a mouse model of lupus through a toll-like receptor 7-dependent mechanism. Immunology.

[111] Desnues B, Macedo AB, Roussel-Queval A, Bonnardel J, Henri S, Demaria O, Alexopoulou L (2014). TLR8 on dendritic cells and TLR9 on B cells restrain TLR7-mediated spontaneous autoimmunity in C57BL/6 mice. Proc Natl Acad Sci U S A.

[112] Subramanian S, Tus K, Li QZ, Wang A, Tian XH, Zhou J, Liang C, Bartov G, McDaniel LD, Zhou XJ, Schultz RA, Wakeland EK (2006). A Tlr7 translocation accelerates systemic autoimmunity in murine lupus. Proc Natl Acad Sci U S A.

[113] Deane JA, Pisitkun P, Barrett RS, Feigenbaum L, Town T, Ward JM, Flavell RA, Bolland S (2007). Control of toll-like receptor 7 expression is essential to restrict autoimmunity and dendritic cell proliferation. Immunity.

[114] Maeda K, Mehta H, Drevets DA, Coggeshall KM (2010). IL-6 increases B-cell IgG production in a feed-forward proinflammatory mechanism to skew hematopoiesis and elevate myeloid production. Blood.

[115] Bandeira F, Lent R, Herculano-Houzel S (2009). Changing numbers of neuronal and non-neuronal cells underlie postnatal brain growth in the rat. Proc Natl Acad Sci U S A.

[116] Thion MS, Garel S (2017). On place and time: microglia in embryonic and perinatal brain development. Curr Opin Neurobiol.

[117] Alliot F, Godin I, Pessac B (1999). Microglia derive from progenitors, originating from the yolk sac, and which proliferate in the brain. Brain Res Dev Brain Res.

[118] Cunningham CL, Martinez-Cerdeno V, Noctor SC (2013). Microglia regulate the number of neural precursor cells in the developing cerebral cortex. J Neurosci.

[119] Voogdt Carlos G.P., van Putten Jos P.M. (2016). The Evolution of the Toll-Like Receptor System. The Evolution of the Immune System.

[120] Mottahedin A, Joakim Ek C, Truve K, Hagberg H, Mallard C (2019). Choroid plexus transcriptome and ultrastructure analysis reveals a TLR2-specific chemotaxis signature and cytoskeleton remodeling in leukocyte trafficking. Brain Behav Immun.

